# Comparison of the Pig Breeds in the Small Intestinal Morphology and Digestive Functions at Different Ages

**DOI:** 10.3390/metabo13010132

**Published:** 2023-01-15

**Authors:** Yating Cheng, Sujuan Ding, Md. Abul Kalam Azad, Bo Song, Xiangfeng Kong

**Affiliations:** 1 Key Laboratory of Agro-Ecological Processes in Subtropical Region, Hunan Provincial Key Laboratory of Animal Nutritional Physiology and Metabolic Process, National Engineering Laboratory for Pollution Control and Waste Utilization in Livestock and Poultry Production, Institute of Subtropical Agriculture, Chinese Academy of Sciences, Changsha 410125, China; 2 College of Advanced Agricultural Sciences, University of Chinese Academy of Sciences, Beijing 100008, China

**Keywords:** digestive function, morphology, nutrient transporter, pig genotype, small intestine

## Abstract

The small intestine is the main site for digestion and absorption of nutrients, and the development of the small intestine can be affected by several factors, such as diet composition, age, and genotype. Thus, this study aimed to compare the small intestinal morphology and digestive function differences at different ages of three pig breeds. Thirty litters of newborn Taoyuan black (TB), Xiangcun black (XB), and Duroc (DR) piglets (ten litters per breed) were selected for this study. Ten piglets from each breed were selected and sampled at 1, 10, 21, and 24 days old. The results showed that the TB and XB piglets had lower growth but had higher lactase and maltase activities in the jejunum compared with the DR piglets, while most of the digestive enzyme activities in the ileum were higher in the DR piglets at different ages. The expression levels of nutrient transporters, mainly including amino acids, glucose, and fatty acids transporters, differed in the jejunum at different ages among three pig breeds and were higher in the DR piglets at 1 day old and XB piglets at 24 days old. Collectively, these findings suggest that the phenotypic differences in the growth, intestinal morphology, and digestive function among the three pig breeds mainly resulted from the differences in digestive enzymes and nutrient transporters in the intestine.

## 1. Introduction

The early growth stage of a piglet is the crucial period of the growth and development in swine production, especially for the growth and development of the stomach and intestine [1]. The development of the gastrointestinal tract during the early growth stage influences further growth performance and production traits by regulating the digestion and absorption ability [2]. The small intestine plays a vital role in nutrient digestion and absorption and is the major organ for host immunity with strong endocrine and exocrine functions [3]. The development and maturity of the small intestine is influenced by several factors, including diet, growth stage, and other stress factors (i.e., physiological, nutritional, environmental, etc.), and most evidence about the growth and development of the small intestine was conducted on the composition of various diets [4,5]. Moreover, the growth and development of piglets is determined by genetic and environmental factors, and the breed accounts for the main cause [6]. However, little information exists about the effects of breed on the intestinal developmental characteristics and functions of piglets. Thus, deciphering how breed affects intestinal growth and development will pave the way for future research regarding the identification of which factors result in differences in growth and development among breeds.

Duroc (DR) pigs are characterized by a higher growth rate, lean meat, and higher feed efficiency and are widely used in breeding programs as the terminal sires for crossed commercial pigs [7]. Chinese domestic pigs, such as Taoyuan black (TB) pigs, are characterized by their higher stress resistance and roughage feeding tolerance while having a lower growth rate and lean meat [8]. Xiangcun black (XB) pigs are the cross-bred of these two (TB and DR) pigs breeds and have the combined advantages of these two pig breeds. Previously, pigs’ genotypes were characterized by different feed efficiency and varied in different ages [9]. Additionally, pigs’ genotypes also affect the survival rate and litter weight gain of piglets by regulating different metabolites in the sow colostrum [10]. Based on the findings mentioned earlier, it can be hypothesized that the divergency in the intestinal morphology and digestive function of different breeds might play a critical role in differential growth and development in Chinese domestic and foreign pigs. Therefore, TB, XB, and DR piglets during suckling and weaning periods were used as the experimental animal to compare the differences in body weight (BW), plasma biochemical parameters, intestine morphology, digestive enzymes activity, and nutrient transporters among the three pig breeds at different ages.

## 2. Materials and Methods

### 2.1. Animals, Experimental Design, and Management

Thirty litters of healthy newborn TB, XB, and DR piglets (10 L per breed) were selected from sows with 2–3 parities and 8–11 L sizes and marked with ear tags. At day 1 (prior to nursing), 10, 21 (weaning), and 24 (day 3 after weaning) of age, one piglet with close to average BW per litter of each breed was selected for sampling (10 piglets per breed, TB and XB piglets were half male and half female, and all the DR piglets were male). The piglets received only sows’ milk during the suckling period. After weaning at 21 days of age, piglets received creep feed, and other feeding management followed the feeding management process of the pig farm for the remaining trial. Piglets were exposed to microorganisms from sows and the surrounding environment. Piglets were housed in individual pen, fed four times daily after weaning (8:00, 12:00, 14:00, and 17:00), and the rearing compartments were temperature-controlled (23–26 °C) and had forced ventilation.

### 2.2. Sample Collection

A total of 30 piglets (10 piglets per breed) were randomly selected and weighed at 1 (at birth), 10, 21, and 24 days of age, 12 h after the last feeding for sample collection. Blood samples from each piglet were collected from the precaval vein into heparinized vacuum tubes (5/10 mL), centrifuged at 3500× *g* for 10 min at 4 °C to collect plasma, and immediately preserved at −80 °C for further analysis of plasma biochemical parameters and plasma-free amino acids. Then, the piglets were euthanized after anaesthetization with sodium pentobarbital (40 mg/kg BW; MTC Pharmaceutical, Cambridge, ON, USA). After euthanizing, the organs and entire intestine were separated, and the isolated intestinal segments were immediately opened lengthwise following the mesentery line. The weight and length of the jejunum and ileum were recorded to calculate the intestinal indexes. The jejunum (10 cm below the duodenum-jejunum junction) and ileum (10 cm above the ileocecal junction) tissues (∼5 cm) were excised, flushed in ice-cold phosphate-buffered saline (PBS), and then scrapped with glass slides. The mucosa scrapings (~2 g) were collected, as described by Christen and Huber [11], quickly frozen into liquid nitrogen, and immediately stored at −80 °C for further analysis of digestive enzyme activity and gene expressions. Additionally, jejunum and ileum segments (~2 cm in length) were washed with PBS to remove intestinal contents, immediately collected into 50 mL centrifuge tubes, and then fixed with 4% paraformaldehyde until processing for the morphology evaluation.

### 2.3. Determination of Plasma Biochemical Parameters

The plasma biochemical parameters, including albumin (ALB), alkaline phosphatase (ALP), alanine aminotransferase (ALT), amylase (AMS), aspartate aminotransferase (AST), urea nitrogen (UN), cholinesterase (CHE), cholesterol (CHO), glucose (GLU), high-density lipoprotein-cholesterol (HDL-C), lactate dehydrogenase (LDH), low-density lipoprotein-cholesterol (LDL-C), ammonia (NH_3_), triglyceride (TG), and total protein (TP) were analyzed using commercially available biochemical kits (Leadman Biochemistry Technology Company, Beijing, China) on Cobas c311 Automatic Biochemical Analyzer (Cobas c311, F. Hoffman-La Roche Ltd., Basel, Switzerland), according to the manufacturers’ instructions.

### 2.4. Determination of Plasma-Free Amino Acids

The plasma samples (800 μL) were mixed with an equal volume of 8% sulfosalicylic acid solution, centrifuged at 8000 × *g* and 4 °C for 10 min, and then filtered through a 0.22 μm membrane to precipitate the protein. Finally, the contents of plasma-free amino acids, including 3-methyl-histidine (3-MH), alanine (Ala), alpha-aminoadipic acid (α-AAA), alpha-aminobutyric acid (α-ABA), arginine (Arg), aspartic acid (Asp), beta-alanine (β-Ala), citrulline (Cit), cystine (Cys), cystathionine (Cysthi), ethanolamine (ETA), glutamic acid (Glu), glycine (Gly), histidine (His), hydroxyl-proline (HYP), isoleucine (Ile), leucine (Leu), L-ornithine (Orn), lysine (Lys), methionine (Met), phenylalanine (Phe), phosphor-serine (P-Ser), proline (Pro), sarcosine (Sar), serine (Ser), taurine (Tau), threonine (Thr), tyrosine (Tyr), and valine (Val) were measured using an Automatic Amino Acid Analyzer (L-8900; Hitachi, Tokyo, Japan).

### 2.5. Analysis of Intestinal Morphology

Jejunal and ileal tissues fixed with 4% paraformaldehyde were trimmed, dehydrated, and embedded in paraffin. Slides with 5 μm tissue sections were stained with hematoxylin and eosin (H&E), as previously described by Saqui-Salces et al. [12]. Well-oriented villi and crypts were measured in ten randomly chosen fields per slide at 40 × magnification under the light microscopy (Olympus BX53) using the Case viewer image software (Digital Pathology Company, Budapest, Hungary). Ten representative measures per tissue were used to measure the villus height (VH, from the tip of the villus to the villus-crypt junction) and crypt depth (CD, from the crypt opening to the base), and the VH/CD ratio was calculated.

### 2.6. Analysis of Small Intestinal Digestive Enzyme Activity

Jejunal and ileal mucosa samples were thawed on ice. Mucosa (~1 g) was extracted by adding ice-cold PBS to mucosa 9 times (1 g: 9 mL), vortexing, and then centrifuging at 3000 × *g* and 4 °C for 15 min. The activities of amylase, chymase, invertase, lactase, lipase, maltase, and trypsin were determined using commercial kits (Shanghai Huyu, Shanghai, China) on a Multiscan Spectrum Spectrophotometer (Infinite M200 Pro; Tecan, Männedorf, Switzerland), following the manufacturer’s instructions.

### 2.7. Analysis of Nutrient Transporter Gene Expression

The nutrient transporter gene expressions in the small intestine were determined by a real-time polymerase chain reaction (RT-PCR), as previously described by Azad et al. [13]. The total RNA from jejunal and ileal tissues was isolated using the TRIzol Reagent (Invitrogen, Carlsbad, CA, USA) following the manufacturer’s instructions. Extracted total RNA concentration was determined using a NanoDrop ND-2000 instrument (Thermo Fisher Scientific, Waltham, MA, USA), and 500 ng of RNA was reverse transcribed to cDNA using PrimeScript RT Reagent Kit with gDNA Eraser (TaKaRa Biotechnology Co., Ltd., Dalian, China). PCR assays were conducted using Premix Ex TaqTM kits (TaKaRa Biotechnology Co., Ltd., Dalian, China). The PCR cycling conditions were as follows: initial denaturation at 95 °C for 30 s, followed by 40 cycles of denaturation at 95 °C for 5 s and annealing at 60 °C for 30 s, and a final extension at 72 °C for 30 s. The glyceraldehyde-3-phosphate dehydrogenase (GAPDH) amplification was used to normalize the expression for each sample of the target genes. The relative gene expression was calculated using the 2^−ΔΔCt^ method, as described by Rocha et al. [14]. The primer sequences for pigs were designed based on the GenBank database in NCBI (Supplementary Table S1).

### 2.8. Statistical Analysis

The normal distribution of the experimental data was confirmed by the Shapiro-Wilk test before assessing differences among the different pig breeds. The data were analyzed by one-way analysis of variance (ANOVA) using the SPSS 22.0 software (IBM Corporation, 2014; Chicago, IL, USA). The mean values of the different experimental groups were compared by Tukey’s post hoc test. The individual piglets were considered as the experimental unit. All data are presented as mean values with their pooled SEM, unless otherwise mentioned. Differences were considered statistically significant when *p* < 0.05 and 0.05 ≤ *p* ≤ 0.10 were considered a trend.

## 3. Results

### 3.1. Differences in Body Weight and Intestinal Indexes of Piglets

The BW and intestinal indexes of piglets are presented in Table 1. The ileum length index of XB piglets was lower (*p* < 0.05) compared with the TB piglets at 1 day old. Compared with the DR piglets, the XB and TB piglets had lower (*p* < 0.05) BW, whereas the XB piglets had higher (*p* < 0.05) jejunum weight and ileum weight and length index at 10 days old. At 21 days old, the BW of XB piglets was lower (*p* < 0.05) and the ileum length index was higher (*p* < 0.05) compared with the TB and DR piglets, whereas the jejunum length index of TB and XB piglets was higher (*p* < 0.05) compared with the DR piglets. At 24 days old, the BW of TB and XB piglets was lower (*p* < 0.05) compared with the DR piglets. In addition, the jejunum and ileum length indexes of XB piglets were higher (*p* < 0.05) compared with the TB and DR piglets.

### 3.2. Differences in Plasma Biochemical Parameters of Piglets

The plasma biochemical parameters of three breeds of piglets at different ages are presented in Table 2. The plasma levels of ALB, CHO, GLU, HDL-C, LDL-C, and TG were lower (*p* < 0.05), whereas AST and UN were higher (*p* < 0.05) in the TB and XB piglets compared with the DR piglets at 1 day old. Moreover, the plasma levels of NH_3_ and TP were higher (*p* < 0.05) in the XB piglets compared with the DR piglets, and LDH was lower (*p* < 0.05) in the TB piglets compared with the DR and XB piglets at 1 day old. At 10 days old, the plasma levels of CHO, LDL-C, TP, and UN were higher (*p* < 0.05), whereas CHE was lower (*p* < 0.05) in the XB piglets compared with the DR piglets. In addition, the plasma level of TG was lower (*p* < 0.05) in the TB piglets compared with the DR piglets at 10 days old. At 21 days old, the plasma level of ALT was lower (*p* < 0.05), whereas HDL-C was higher (*p* < 0.05) in the TB piglets compared with the DR piglets. Moreover, the plasma level of UN was higher (*p* < 0.05) in the XB piglets compared with the DR piglets, and NH_3_ was higher (*p* < 0.05) in the TB piglets compared with the XB and DR piglets at 21 days old. At 24 days old, the plasma levels of CHO in the TB piglets, AST in the XB piglets, and HDL-C and LDL-C in the TB and XB piglets were higher (*p* < 0.05) when compared with the DR piglets.

### 3.3. Differences in Plasma-Free Amino Acids of Piglets

The plasma-free amino acid contents of three breeds of piglets at different ages are presented in Table 3. The plasma Phe, P-Ser, and α-AAA contents were higher (*p* < 0.05), whereas Thr and Gly contents were lower (*p* < 0.05) in the TB and XB piglets compared with the DR piglets at 1 day old. Compared with the TB piglets, plasma Glu, His, Leu, Lys, and Val contents in the XB and DR piglets; α-ABA content in the XB piglets; and Cit, Pro, and Tyr contents in the DR piglets were lower (*p* < 0.05) at 1 day old. Moreover, the plasma Asp content was lower (*p* < 0.05) in the TB and DR piglets, and Ile content was higher (*p* < 0.05) in the DR piglets at 1 day old when compared with the XB piglets.

At 10 days old, the plasma HYP, Pro, Sar, and Ser contents in the TB piglets; Arg, Lys, and Val contents in the TB and XB piglets; and His and Orn contents in the XB piglets were higher (*p* < 0.05) compared with the DR piglets. Compared with the TB piglets, the plasma α-ABA content was higher (*p* < 0.05), whereas α-AAA content was lower (*p* < 0.05) in the XB and DR piglets. Moreover, the plasma 3-MH and P-Ser contents were higher (*p* < 0.05), and the Glu content was lower (*p* < 0.05) in the XB piglets compared with the DR and TB piglets.

At 21 days old, the plasma Ala, Arg, Cit, HYP, Lys, Met, Orn, Pro, Tau, Thr, Tyr, and α-AAA contents were lower (*p* < 0.05), while Cysthi and α-ABA contents were higher (*p* < 0.05) in the XB and DR piglets compared with the TB piglets. The plasma Asp and His contents in the TB and XB piglets and Ser content in the TB piglets were higher (*p* < 0.05), while Cys content in the TB and XB piglets was lower (*p* < 0.05) compared with the DR piglets. Moreover, the plasma P-Ser and 3-MH contents were higher (*p* < 0.05), while Glu content was lower (*p* < 0.05) in the XB piglets compared with the TB and DR piglets.

At 24 days old, the plasma Asp and α-AAA contents were lower (*p* < 0.05), while theGlu and HYP contents were higher (*p* < 0.05) in the TB and XB piglets compared with the DR piglets. Compared with the XB piglets, the plasma Cit, Orn, and P-Ser contents were lower (*p* < 0.05), while the Car content was higher (*p* < 0.05) in the TB and DR piglets. Moreover, the plasma Arg content in XB piglets was higher (*p* < 0.05) compared with the DR piglets, whereas the ETA and HYP contents were lower (*p* < 0.05) in the XB and DR piglets compared with the TB piglets.

### 3.4. Differences in Intestinal Morphology of Piglets

**Figure 1:**
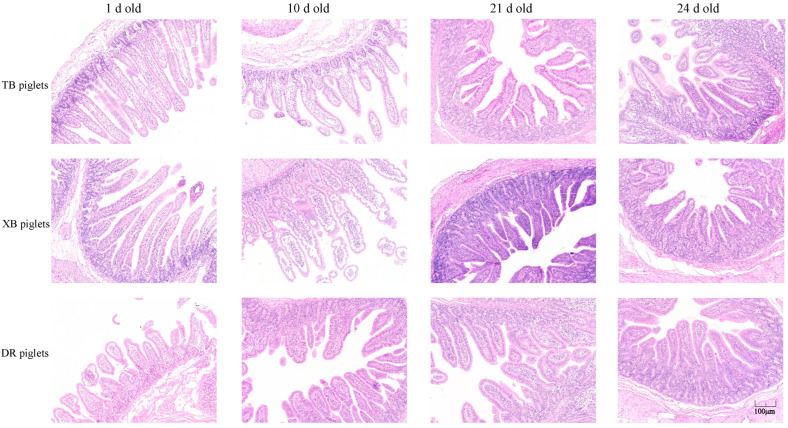
Comparison of jejunal histological appearance of three breeds of piglets at different ages (*n* = 10). Sections were stained by hematoxylin and eosin. TB piglets, Taoyuan black piglets; XB piglets, Xiangcun black piglets; DR piglets, Duroc piglets. Scale bars, 40 ×.

The jejunal morphology of the three breeds of piglets at different ages is presented in Figure 1 and Table 4. At 1 day old, the VH and VH/CD were higher (*p* < 0.05) in the TB and XB piglets compared with the DR piglets. Additionally, XB piglets had a higher (*p* < 0.05) VH compared with the TB piglets at 1 day old. At 21 days old, the VH and CD in the TB and XB piglets were lower (*p* < 0.05) compared with the DR piglets. However, there were no significant differences (*p* > 0.05) in the jejunal VH, CD, and VH/CD between the three pig breeds at 10 and 24 days old (Table 4). Moreover, there were no visible changes in the jejunum of piglets among the different pig breeds at different ages (Figure 1).

**Figure 2:**
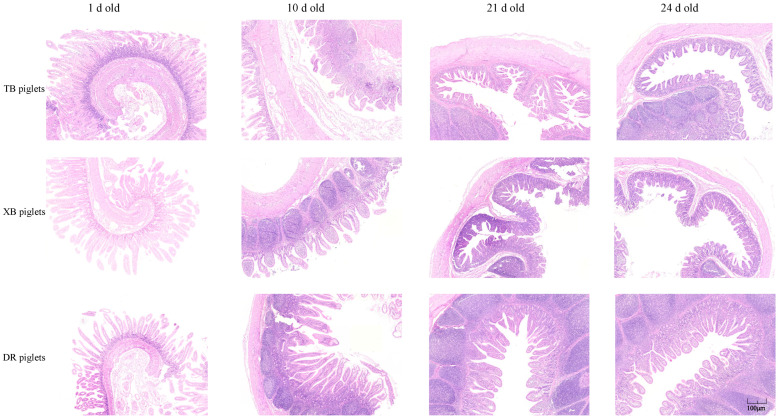
Comparison of ileal histological appearance of three breeds of piglets at different ages (*n* = 10). Sections were stained by hematoxylin and eosin. TB piglets, Taoyuan black piglets; XB piglets, Xiangcun black piglets; DR piglets, Duroc piglets. Scale bars, 40 ×.

The ileal morphology of the three breeds of piglets at different ages is presented in Figure 2 and Table 5. At 1 day old, the VH in the XB piglets and VH/CD in the TB and XB piglets were higher (*p* < 0.05) compared with the DR piglets. At 10 days old, the CD in the TB piglets was lower (*p* < 0.05) compared with the DR piglets. However, there were no significant differences (*p* > 0.05) in the ileal VH, CD, and VH/CD between the three pig breeds at 21 and 24 days old (Table 5). Moreover, there were no visible changes in the ileum of piglets among the different breeds at different ages (Figure 2).

### 3.5. Differences in Digestive Enzyme Activities of Piglets in Small Intestinal Mucosa

The jejunal digestive enzyme activities of three breeds of piglets at different ages are presented in Table 6. At 1 day old, the amylase activity in the TB and XB piglets was lower (*p* < 0.05), whereas the lactase and maltase activities in the TB and XB piglets and invertase activity in the XB piglets were higher (*p* < 0.05) compared with the DR piglets. In addition, the trypsin activity in the XB and DR piglets was lower (*p* < 0.05) compared with the TB piglets. At 10 days old, the amylase activity was higher (*p* < 0.05), and the lipase and trypsin activities were lower (*p* < 0.05) in the XB and DR piglets compared with the TB piglets. Moreover, the chymase activity in the TB and XB piglets was lower (*p* < 0.05) compared with the DR piglets. At 21 days old, the lactase and maltase activities were higher (*p* < 0.05), while the amylase activity was lower (*p* < 0.05) in the TB and XB piglets compared with the DR piglets. The trypsin activity was higher (*p* < 0.05) but the invertase activity was lower (*p* < 0.05) in the XB piglets compared with the TB and DR piglets, and the lipase activity was lower (*p* < 0.05) compared with the DR piglets. Moreover, the lipase activity was lower (*P* < 0.05) in the XB piglets compared with the DR piglets, whereas the trypsin activity was higher (*P* < 0.05) compared with the TB and DR piglets. At 24 days old, the maltase activity was higher (*p* < 0.05) while the amylase activity was lower (*p* < 0.05) in the TB and XB piglets compared with the DR piglets. The invertase and lactase activities were higher (*p* < 0.05) in the XB piglets compared with the DR piglets. Moreover, the trypsin activity in the XB piglets was higher (*p* < 0.05) compared with the TB and DR piglets.

The ileal digestive enzyme activities of three breeds of piglets at different ages are presented in Table 7. Compared with the DR piglets, the amylase, chymase, invertase, lactase, lipase, maltase, and trypsin activities were lower (*p* < 0.05) in the TB and XB piglets at 1 day old. Moreover, the lactase and maltase activities were lower (*p* < 0.05) in the TB piglets compared with the XB piglets at 1 day old. At 10 days old, the chymase, lactase, lipase, and maltase activities in the TB piglets were lower (*p* < 0.05) compared with the DR and XB piglets. At 21 days old, the lactase and maltase activities were lower (*p* < 0.05) in the TB and XB piglets compared with the DR piglets. Compared with the TB and DR piglets, the amylase, invertase, and trypsin activities were lower (*p* < 0.05) in the XB piglets. Moreover, the amylase activity was lower (*p* < 0.05) in the XB piglets compared with the DR piglets. At 24 days old, the chymase, lactase, lipase, and maltase activities were lower (*p* < 0.05) in the TB and XB piglets compared with the DR piglets. The amylase, invertase, and trypsin activities were lower (*p* < 0.05) in the XB piglets compared with the TB and DR piglets. Moreover, the lipase activity was higher (*P* < 0.05), whereas the maltase activity was lower (*P* < 0.05) in the TB piglets compared with the XB piglets at 24 days old.

### 3.6. Differences in Intestinal Nutrient Transporter Gene Expressions of Piglets

**Figure 3:**
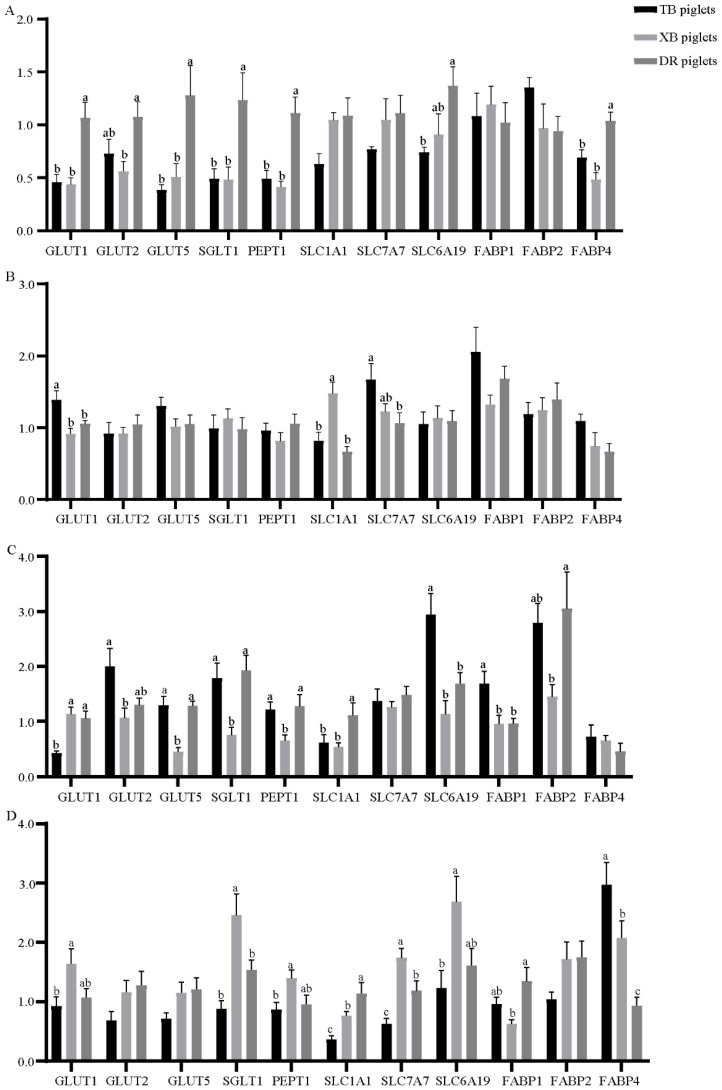
Differences in the jejunal nutrient transporters of piglets at 1 day old (**A**), 10 days old (**B**), 21 days old (**C**), and 24 days old (**D**) (*n* = 8). ^a,b,c^ Different letters indicate a significant difference (*p* < 0.05). TB piglets, Taoyuan black piglets; XB piglets, Xiangcun black piglets; DR piglets, Duroc piglets. *GLUT*, glucose transporter; *SGLT1*, sodium-glucose linked transporter 1; *PEPT1*, peptide transporter 1; *SLC1A1*, solute carrier family 1 member 1; *SLC7A7*, solute carrier family 7 member 7; *SLC6A19*, solute carrier family 6 member 19; *SLC1A5*, solute carrier family 1 member 5; *SLC7A9*, solute carrier family 7 member 9; *FABP*, fatty-acid-binding protein.

The jejunal nutrient transporters expression of the three breeds of piglets at different ages is presented in Figure 3. At 1 day old (Figure 3A), the glucose transporter 1 (*GLUT1*), glucose transporter 5 (*GLUT5*), sodium-glucose linked transporter 1 (*SGLT1*), peptide transporter 1 (*PEPT1*), and fatty-acid-binding protein 4 (*FABP4*) expressions were down-regulated (*p* < 0.05) in the TB and XB piglets compared with the DR piglets. Furthermore, the glucose transporter 2 (*GLUT2*) expression in the XB piglets and solute carrier family 6 member 19 (*SLC6A19*) expression in the TB piglets were down-regulated (*p* < 0.05) compared with the DR piglets. At 10 days old (Figure 3B), the *GLUT1* expression was up-regulated (*p* < 0.05) in the TB piglets compared with the XB and DR piglets. Compared with the XB piglets, the solute carrier family 7 member 7 (*SLC7A7*) expression was down-regulated (*p* < 0.05) in the TB and DR piglets. Moreover, the solute carrier family 1 member 1 (*SLC1A1*) expression was up-regulated (*p* < 0.05) in the TB piglets compared with the DR piglets. At 21 days old (Figure 3C), the *GULT5*, *SGLT1*, and *PEPT1* expressions were down-regulated (*p* < 0.05) in the XB piglets compared with the TB and DR piglets. The *SLC7A7* expression of the TB and XB piglets and *FABP2* expression of the XB piglets were down-regulated (*p* < 0.05) compared with the DR piglets. Compared with the TB piglets, the *GULT1* expression was up-regulated (*p* < 0.05), while *SLC6A19* expression was down-regulated (*p* < 0.05) in the XB and DR piglets. At 24 days old (Figure 3D), the *GLUT1*, *PEPT1*, and *SLC6A19* expressions of the TB piglets and *SGLT1* and *SLC7A7* expressions of the TB and DR piglets were down-regulated (*p* < 0.05) compared with the XB piglets. The *FABP4* expression of the TB piglets was up-regulated (*p* < 0.05) compared with the DR and XB piglets, whereas the *SLC1A1* expression of the TB and XB piglets was down-regulated (*p* < 0.05) compared with the DR piglets.

**Figure 4:**
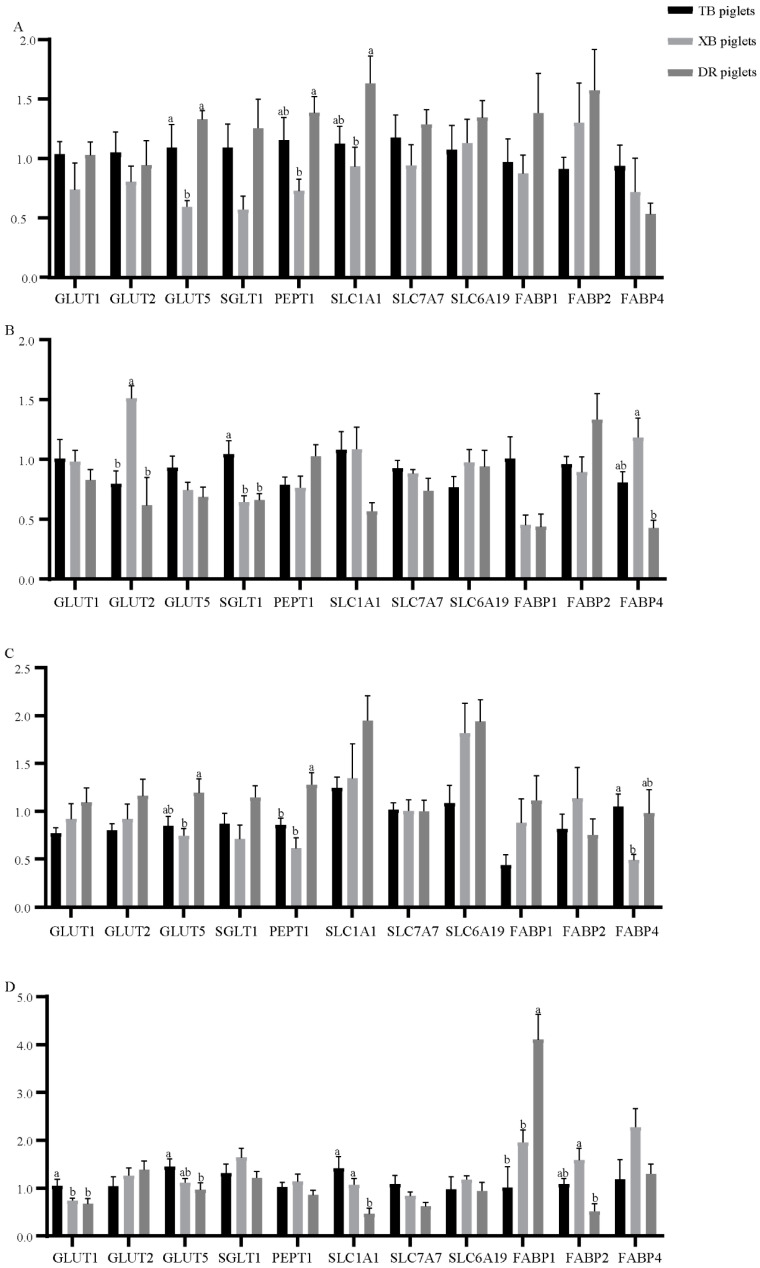
Differences in the ileal nutrient transporters of piglets at 1 day old (**A**), 10 days old (**B**), 21 days old (**C**), and 24 days old (**D**) (*n* = 8). ^a,b^ Different letters indicate a significant difference (*p* < 0.05). TB piglets, Taoyuan black piglets; XB piglets, Xiangcun black piglets; DR piglets, Duroc piglets. *GLUT*, glucose transporter; *SGLT1*, sodium-glucose linked transporter 1; *PEPT1*, peptide transporter 1; *SLC1A1*, solute carrier family 1 member 1; *SLC7A7*, solute carrier family 7 member 7; *SLC6A19*, solute carrier family 6 member 19; *SLC1A5*, solute carrier family 1 member 5; *SLC7A9*, solute carrier family 7 member 9; *FABP*, fatty-acid-binding protein.

The ileal nutrient transporters expression of the three breeds of piglets at different ages is presented in Figure 4. At 1 day old (Figure 4A), the *GLUT5* expression of the XB piglets was down-regulated (*p* < 0.05) compared with the DR and TB piglets. The *PEPT1* and *SLC1A1* expressions of the XB piglets were down-regulated (*p* < 0.05) compared with the DR piglets. At 10 days old (Figure 4B), the *SGLT1* expression of the TB piglets was up-regulated (*p* < 0.05) compared with the DR and XB piglets. The *GLUT2* expression of the DR and TB piglets and *FABP4* expression of the DR piglets were down-regulated (*p* < 0.05) compared with the XB piglets. At 21 days old (Figure 4C), the *GLUT5* expression of the XB piglets and *PEPT1* expression of the TB and XB piglets were down-regulated (*p* < 0.05) compared with the DR piglets. Compared with the XB piglets, the *FABP4* expression was up-regulated (*p* < 0.05) in the TB piglets. At 24 days old (Figure 4D), the *GLUT1* expression of the DR and XB piglets and *GLUT5* expression of the DR piglets were down-regulated (*p* < 0.05) compared with the TB piglets. The *SLC1A1* expression of the TB and XB piglets and *FABP2* expression of the XB piglets were up-regulated (*p* < 0.05), while the *FABP1* expression of the TB and XB piglets was down-regulated (*p* < 0.05) compared with the DR piglets.

## 4. Discussion

The development and function of the small intestine can influence the growth and development of the pigs. Thus, the present study was conducted to comprehensively compare the small intestinal morphology and function and its dynamic changes throughout piglets’ growth stages. In order to investigate the original differences in the small intestine among three pig breeds in practical pig production, we did not consider the maternal factors, such as milk intake and composition, which are the limitations of this study. However, we reported these influences in our other studies (unpublished data). Our findings showed that BW, jejunal, and ileal indexes were significantly different among three pig breeds at 1, 10, 21, and 24 days old, which were in response to the changes in digestive enzyme activities and nutrient transporter gene expressions in the small intestine of piglets.

The weight and length of the intestine are important indicators to measure the development of the small intestine [15]. In the present study, the BW of TB and XB piglets was lower, while their jejunum and ileum weight and length indexes were higher at different age stages. These results indicate that the growth of domestic pig breeds was slower than the foreign breeds, while the proportion of the intestine of domestic pig breeds was higher and possessed strong development and digestive capacity. Poor feeding management, nutrition sources, and environment contribute to tolerance to roughage feeding of Chinese domestic pigs [16]. Thus, the higher relative weight of the intestine requires a higher proportion of body energy expenditure and oxygen consumption, which may be associated with the reduced growth efficiency of Chinese domestic and crossed pigs in the present study. The findings are also in agreement with Wang et al. [17], who reported that there was no significant increase in the growth performance of the pigs with longer lengths of the small intestine, which may be partly due to the higher energy expenditure rate of the small intestine. Furthermore, the relative weight and length of the small intestine of DR piglets decreased from 1 to 21 days old and then increased after weaning. It was consistent with Elefson et al. [18], who reported that the relative weight of the majority of visceral organs decreased with the increase in piglet’s age within the first 3–7 days after birth in the crossbred pigs (involved in various crossbred, including Large White, Yorkshire, Landrace, and DR). However, this trend was not observed in the TB and XB piglets. This discrepancy may be correlated with the breeds, and further studies are needed to reveal the specific reason.

Plasma biochemical parameters and plasma-free amino acid content can reflect the metabolism and growth of the body [19]. The ALT and AST are important transaminases in the amino acid metabolism, and a higher concentration means a stronger amino acid metabolism [20]. The TP represents protein metabolism, and the higher TP concentration is associated with stronger protein synthesis than the catabolism of the body [21]. TG is the total triglyceride in the plasma, and the higher concentration means the weaker fat deposition [22]. Moreover, HDL-C and LDL-C are involved in the transportation of the lipid, and CHE is the crucial enzyme in lipid metabolism [23]. Our findings showed that the plasma levels of TG, LDL-C, and CHE were lower in the TB and XB piglets at 1 and 10 days old, while those were higher at 21 and 24 days old than in the DR piglets. These results suggest that the lipid synthesis and transportation in the DR piglets declined by the weaning stress, while the Chinese domestic and crossed pig breeds, especially the TB pigs, have stronger stress resistance to the weaning stress. These findings were consistent with Song et al. [24], who evaluated the capacity of immunological stress on the Chinese domestic breed (Laiwu), crossbred (Lulai), and exotic breed (Yorkshire) and found that Laiwu pigs had stronger resistance to immunological stress among the three pig breeds. Moreover, the plasma TP, UN, and AST levels in the XB and TB piglets and several plasma-free amino acids (including Glu, Lys, HYP, Phe, and Pro) contents in the TB and XB piglets were higher than in the DR piglets between 1–24 days old. Plasma amino acid contents can represent the amino acid metabolism in the whole body, but the composition may be affected by several factors, such as diet and time [25]. McGilvray et al. [26] found that pigs during the immune system stimulation induced by the lipopolysaccharide had reduced plasma Lys, Phe, and Ile contents, which may contribute to a decrease in whole-body protein synthesis or decreased catabolism of these amino acids. Taken together, our results suggest that the TB and XB piglets exhibit a strong ability for whole-body protein synthesis and amino acid metabolism.

Intestinal morphology plays an important role in nutrient digestion and absorption in mammals. The VH, CD, and VH/CD can directly reflect enterocyte proliferation and affect nutrient absorption and transportation in the small intestine. During the early growth stage of piglets, changing diet types and weaning stress are highly associated with the development of the small intestine. The higher VH and lower CD of the small intestine indicate the better absorptive and digestive ability of the small intestine [27]. In the present study, the jejunal and ileal VH and VH/CD were higher in the TB piglets at 1 day old, whereas the ileal CD at 10 days old and jejunal VH and CD at 21 days old were higher in the DR piglets. These results indicate that the nutrient absorptive ability of the jejunum and ileum was stronger in the TB and DR piglets than the XB piglets, and it may be related to the maturation of the intestine. In addition, the jejunal and ileal VH gradually decreased, and the CD increased in the TB and XB piglets after weaning, while DR piglets had the opposite trend. However, a previous study by Walsh et al. [28] found a reduction in VH and an increase in CD in the duodenum and jejunum of crossbred (Large white × Landrace) piglets after weaning. This discrepancy might have resulted from the diet types and breeds of pigs in the present study.

Digestive enzymes are secreted to the brush border membrane to facilitate nutrient absorption, and their activities in the small intestine can reflect the digestive ability of the piglets [29]. Intestinal digestive enzymes are involved in the digestion of carbohydrates, proteins, and fat. Carbohydrates are the main energy sources of the piglets; thus, the disaccharidase activities were higher than the trypsin and lipase in the jejunum and ileum [30]. Disaccharidase activity is usually used to represent the maturity of the intestine, especially by the activities of lactase and maltase [31]. In the present study, the lactase and maltase activities at 1 and 21 days old and maltase activity at 24 days old were higher in the jejunum of the TB and XB piglets than those in the DR piglets, suggesting that the TB and XB piglets have stronger jejunal digestive abilities, and the development and maturity of the jejunum was better. In addition, those activities in the ileum of the DR piglets were higher at different ages than in the XB piglets, which may be related to the ileal brake, which means that the undigested nutrients reach the ileum and induce the activation of the ileum to influence the digestive process [32].

Digested nutrients, such as glucose, lipid, and amino acids in the small intestine are absorbed by various nutrient transporters located at the enterocyte brush borders [33]. The *GLUT1*, *GLUT2* (mediated glucose), and *GLUT5* (mediated fructose) play important roles in the transport process of monosaccharides in the mammalian intestine, and *SGLT1* mediates the absorption of D-glucose and D-galactose in the intestine [34]. The *PEPT1*, *SLC1A1*, *SLC7A7*, and *SLC6A19* have been identified as the major intestinal transporters for peptides, acidic, basic, and neutral amino acids, respectively [35], while *FABP1*, *FABP2*, and *FABP4* are the major intestinal transporters for lipids [36]. Our results showed that the differences in amino acid transporter expressions mainly appeared in the jejunum. The differences might be related to the absorption process in the jejunum. Similarly, Vigors et al. [37] reported that pigs with a low residual feed intake were associated with higher total intestinal tract digestibility and had higher expression levels of fatty acid transporters. These results were in agreement with the DR pigs in the present study, characterized by lower residual feed intake, and had higher expressions of different fatty acid transporter in the jejunum at different ages. Furthermore, the expression levels of saccharide and amino acid transporters in the jejunum were higher in the TB and DR piglets than in the XB piglets from 1 to 21 days old, while those were decreased at 24 days old. These results might be related to the resistance to weaning stress ability in the XB pigs. In addition, the nutrient transporter expression trends were similar to the VH in the jejunum and ileum. These results were consistent with Wang et al. [38], who reported that the expressions of amino acid transporters were positively associated with the ileal VH.

## 5. Conclusions

Our results indicate that the DR piglets have exhibited higher BW gain, lipid metabolism ability before weaning, and ileal digestive enzyme activities, while the TB and XB piglets have a higher proportion of intestinal index, nitrogen metabolism ability, and higher jejunal lactase and maltase activities. Furthermore, the intestinal morphology, nutrient transporters, and enzyme activities changed with the piglet’s ages, which might be correlated with the differences in milk composition and intake; however, further studies are needed to explore this issue. Unfortunately, the effects of the milk intake and milk composition, which might be related to the piglet’s digestion capacity, were not investigated in this study. Moreover, the dry matter intake of the piglets, which might be associated with the difference in milk intake and milk composition, feed intake, and access of piglets to maternal feces, was also not evaluated. However, these effects need to be considered in future studies for a better understanding of the coprophagy impacts on the growth, development, and immunocompetence of piglets. In summary, the Chinese domestic and crossed pig breeds had lower growth indicators and stronger intestinal growth and anti-stress ability during the suckling and weaning periods. These findings have important implications for understanding and fully utilizing the advantages of foreign and Chinese domestic pigs in pig breeding.

## Figures and Tables

**Table 1:** Differences in body weight and intestinal indexes of piglets at different ages.

Item	TB Piglets	XB Piglets	DR Piglets	SEM	*p*-Values
1 days old					
Body weight (kg)	1.50	1.39	1.82	0.08	0.075
Jejunum (g/kg)	19.12	18.79	17.49	0.94	0.769
Ileum (g/kg)	19.41	17.04	18.04	1.16	0.715
Jejunum length index (cm/kg)	129.27	116.86	109.60	5.81	0.391
Ileum length index (cm/kg)	137.86 ^a^	89.61 ^b^	109.84 ^ab^	6.47	0.006
10 days old					
Body weight (kg)	2.65 ^b^	2.63 ^b^	3.86 ^a^	0.16	<0.001
Jejunum (g/kg)	15.32 ^ab^	17.81 ^a^	14.64 ^b^	0.50	0.020
Ileum (g/kg)	17.75 ^ab^	20.42 ^a^	16.82 ^b^	0.55	0.016
Jejunum length index (cm/kg)	88.54	94.28	75.18	3.69	0.092
Ileum length index (cm/kg)	86.68 ^ab^	91.29 ^a^	70.82 ^b^	3.21	0.018
21 days old					
Body weight (kg)	5.06 ^a^	3.42 ^b^	6.01 ^a^	0.26	<0.001
Jejunum (g/kg)	17.67	18.96	14.66	1.13	0.305
Ileum (g/kg)	20.07	20.09	16.75	0.86	0.192
Jejunum length index (cm/kg)	70.66 ^a^	86.33 ^a^	51.80 ^b^	3.81	<0.001
Ileum length index (cm/kg)	69.35 ^b^	91.42 ^a^	51.66 ^b^	4.52	<0.001
24 days old					
Body weight (kg)	4.37 ^b^	3.22 ^c^	5.74 ^a^	0.24	<0.001
Jejunum (g/kg)	15.03	17.91	15.51	0.79	0.290
Ileum (g/kg)	15.25	19.55	17.80	0.74	0.055
Jejunum length index (cm/kg)	68.95 ^b^	91.68 ^a^	55.48 ^b^	4.08	<0.001
Ileum length index (cm/kg)	68.95 ^b^	91.68 ^a^	55.50 ^b^	4.08	<0.001

Data are presented as means with their pooled SEM (*n* = 10). ^a,b,c^ Mean values with different letters indicate significant difference (*p* < 0.05). TB piglets, Taoyuan black piglets; XB piglets, Xiangcun black piglets; DR piglets, Duroc piglets.

**Table 2:** Differences in plasma biochemical parameters of piglets at different ages.

Item	TB Piglets	XB Piglets	DR Piglets	SEM	*p*-Values
1 days old					
ALB (g/L)	13.91 ^b^	15.73 ^b^	25.30 ^a^	1.28	<0.001
ALP (U/L)	1850.60	1820.60	2518.60	230.87	0.391
ALT (U/L)	29.77	42.07	38.53	2.40	0.088
AMS (U/L)	2129.00	1797.20	1985.60	99.39	0.407
AST (g/L)	124.70 ^a^	156.40 ^a^	61.75 ^b^	10.16	<0.001
CHE (mmol/L)	362.80	356.60	445.20	17.91	0.074
CHO (mmol/L)	1.57 ^b^	1.76 ^b^	4.11 ^a^	0.25	<0.001
GLU (mmol/L)	4.15 ^b^	4.77 ^b^	6.53 ^a^	0.28	<0.001
HDL-C (mmol/L)	0.57 ^b^	0.63 ^b^	1.34 ^a^	0.08	<0.001
LDH (U/L)	441.40 ^b^	650.40 ^a^	700.20 ^a^	36.81	0.005
LDL-C (mmol/L)	0.87 ^b^	1.03 ^b^	2.44 ^a^	0.17	<0.001
NH_3_ (μmol/L)	372.15 ^b^	478.83 ^a^	395.45 ^b^	15.39	0.006
TG (mmol/L)	0.49 ^b^	0.35 ^b^	0.74 ^a^	0.04	<0.001
TP (g/L)	63.99 ^ab^	74.77 ^a^	62.38 ^b^	2.15	0.032
UN (U/L)	5.20 ^a^	5.97 ^a^	2.84 ^b^	0.40	0.001
10 days old					
ALB (g/L)	36.09	37.76	39.92	0.76	0.118
ALP (U/L)	926.80	1403.20	1279.20	90.80	0.080
ALT (U/L)	26.57	30.12	31.89	0.98	0.073
AMS (U/L)	2091.60	2296.00	2645.20	135.66	0.249
AST (g/L)	56.30	61.40	60.70	2.51	0.683
CHE (mmol/L)	577.00 ^ab^	477.60 ^b^	653.80 ^a^	21.94	0.002
CHO (mmol/L)	4.53 ^ab^	6.30 ^a^	4.48 ^b^	0.33	0.029
GLU (mmol/L)	6.26	6.09	6.39	0.17	0.792
HDL-C (mmol/L)	1.35	1.60	1.53	0.05	0.146
LDH (U/L)	686.80	756.60	804.00	28.35	0.243
LDL-C (mmol/L)	3.32 ^ab^	4.78 ^a^	2.97 ^b^	0.29	0.023
NH_3_ (μmol/L)	375.55	428.68	402.72	16.87	0.453
TG (mmol/L)	0.30 ^b^	0.48 ^ab^	0.53 ^a^	0.04	0.018
TP (g/L)	62.52 ^ab^	64.02 ^a^	56.57 ^b^	1.23	0.027
UN (U/L)	3.16 ^ab^	4.60 ^a^	2.62 ^b^	0.30	0.014
21 days old					
ALB (g/L)	48.36	46.87	45.87	1.14	0.682
ALP (U/L)	657.20	573.60	572.80	45.29	0.697
ALT (U/L)	27.33 ^b^	32.17 ^ab^	39.91 ^a^	2.01	0.030
AMS (U/L)	2738.20	1969.40	2178.00	167.20	0.153
AST (g/L)	64.50	87.10	61.50	5.11	0.078
CHE (mmol/L)	625.20	539.20	671.40	28.46	0.158
CHO (mmol/L)	5.07	3.66	3.40	0.35	0.112
GLU (mmol/L)	6.40	6.69	6.28	0.16	0.570
HDL-C (mmol/L)	1.56 ^a^	1.33 ^ab^	1.17 ^b^	0.06	0.020
LDH (U/L)	832.80	816.20	881.60	49.87	0.865
LDL-C (mmol/L)	3.60	2.13	2.02	0.36	0.132
NH_3_ (μmol/L)	377.42 ^a^	258.04 ^b^	213.67 ^b^	19.21	<0.001
TG (mmol/L)	0.48	0.60	0.53	0.03	0.379
TP (g/L)	61.20	61.64	60.02	0.97	0.792
UN (U/L)	4.56 ^ab^	5.23 ^a^	3.18 ^b^	0.30	0.011
24 days old					
ALB (g/L)	46.67	45.33	43.24	0.94	0.335
ALP (U/L)	420.00	366.60	361.20	22.97	0.529
ALT (U/L)	25.21	34.63	27.32	1.98	0.123
AMS (U/L)	2107.00	2236.40	1760.80	105.74	0.167
AST (g/L)	47.10 ^ab^	63.70 ^a^	38.40 ^b^	3.80	0.016
CHE (mmol/L)	633.40	612.60	636.00	26.51	0.930
CHO (mmol/L)	5.69 ^a^	4.58 ^ab^	2.79 ^b^	0.45	0.025
GLU (mmol/L)	5.09	5.70	5.96	0.18	0.115
HDL-C (mmol/L)	1.22 ^a^	1.38 ^a^	0.88 ^b^	0.06	0.002
LDH (U/L)	630.80	724.20	599.80	31.43	0.250
LDL-C (mmol/L)	3.26 ^a^	3.00 ^a^	1.36 ^b^	0.29	0.012
NH_3_ (μmol/L)	179.99	163.45	135.34	11.06	0.055
TG (mmol/L)	0.81	0.67	0.48	0.07	0.148
TP (g/L)	58.97	62.19	60.70	1.26	0.597
UN (U/L)	3.87	4.28	2.75	0.41	0.303

Data are presented as means with their pooled SEM (*n* = 10). ^a,b^ Mean values with different letters indicate significant difference (*p* < 0.05). TB piglets, Taoyuan black piglets; XB piglets, Xiangcun black piglets; DR piglets, Duroc piglets. ALB, albumin; ALP, alkaline phosphatase; ALT, alanine aminotransferase; AMS, amylase; AST, aspartate aminotransferase; CHE, cholinesterase; CHO, cholesterol; GLU, glucose; HDL-C, high-density lipoprotein-cholesterol; LDH, lactate dehydrogenase; LDL-C, low-density lipoprotein-cholesterol; NH_3_, ammonia; TG, triglyceride; TP, total protein; UN, urea nitrogen.

**Table 3:** Differences in plasma-free amino acids of piglets at different ages (μg/mL).

Item	TB Piglets	XB Piglets	DR Piglets	SEM	*p*-Values
1 days old					
3-MH	1.62	1.59	1.00	0.17	0.316
Ala	50.08	53.21	45.66	1.89	0.268
Arg	37.55	14.80	9.30	6.19	0.143
Asp	2.33 ^b^	6.06 ^a^	1.81 ^b^	0.42	<0.001
Car	2.47	2.80	0.50	0.27	0.108
Cit	21.04 ^a^	18.76 ^ab^	15.28 ^b^	0.93	0.033
Cys	8.34	6.62	5.94	0.56	0.198
ETA	0.49	0.61	0.61	0.05	0.479
Glu	71.30 ^a^	24.48 ^c^	48.82 ^b^	4.76	<0.001
Gly	42.63 ^b^	34.74 ^b^	68.73 ^a^	3.63	<0.001
His	18.72 ^a^	12.02 ^b^	10.83 ^b^	1.04	0.002
HYP	11.14	7.57	13.34	1.10	0.095
Ile	10.90 ^ab^	7.69 ^b^	13.74 ^a^	0.84	0.008
Leu	28.49 ^a^	19.28 ^b^	13.89 ^b^	1.70	<0.001
Lys	38.16 ^a^	23.41 ^b^	18.00 ^b^	2.31	<0.001
Met	11.09	6.17	9.38	1.01	0.122
Orn	16.38	14.90	11.63	0.92	0.095
Phe	24.51 ^a^	22.45 ^a^	15.56 ^b^	1.13	0.001
Pro	70.40 ^a^	51.79 ^ab^	41.17 ^b^	4.00	0.006
P-Ser	4.62 ^a^	5.54 ^a^	2.64 ^b^	0.33	0.001
Sar	1.33	0.64	0.45	0.16	0.055
Ser	24.03	21.59	19.09	0.91	0.083
Tau	11.08	12.75	10.39	0.83	0.507
Thr	15.19 ^b^	10.42 ^b^	26.08 ^a^	1.82	<0.001
Tyr	50.57 ^a^	42.62 ^ab^	30.80 ^b^	3.32	0.045
Val	65.37 ^a^	45.54 ^b^	28.96 ^c^	3.73	<0.001
α-AAA	8.95 ^a^	9.34 ^a^	1.13 ^b^	1.10	<0.001
α-ABA	1.45 ^a^	0.79 ^b^	1.24 ^ab^	0.11	0.047
β-Ala	1.26	1.47	1.79	0.11	0.167
10 days old					
3-MH	1.59 ^b^	2.13 ^a^	1.28 ^b^	0.11	0.004
Ala	45.89	36.67	36.49	2.39	0.202
Arg	23.69 ^a^	21.02 ^a^	11.44 ^b^	1.53	0.001
Asp	2.48	2.20	1.53	0.18	0.110
Car	2.98	2.83	0.50	0.30	0.170
Cit	22.67	24.04	17.80	1.13	0.053
Cys	7.49	8.90	8.74	0.27	0.054
Cysthi	1.00	1.29	1.15	0.08	0.350
ETA	0.98	1.13	0.83	0.06	0.165
Glu	50.77 ^a^	13.97 ^b^	52.72 ^a^	3.96	<0.001
Gly	70.58	59.90	69.58	3.07	0.302
His	9.22 ^ab^	9.99 ^a^	6.26 ^b^	0.61	0.025
HYP	26.99 ^a^	23.45 ^ab^	20.82 ^b^	1.03	0.044
Ile	16.29	20.19	18.10	0.77	0.113
Leu	15.99	21.90	16.29	1.27	0.095
Lys	32.34 ^a^	24.55 ^a^	13.17 ^b^	2.14	<0.001
Met	10.43	10.34	9.40	0.53	0.695
Orn	16.26 ^ab^	21.09 ^a^	13.27 ^b^	1.10	0.009
Phe	16.49	16.52	13.93	0.61	0.137
Pro	50.35 ^a^	39.45 ^ab^	27.70 ^b^	3.06	0.004
P-Ser	3.88 ^b^	4.95 ^a^	1.98 ^c^	0.29	<0.001
Sar	1.26 ^a^	0.89 ^ab^	0.33 ^b^	0.11	0.009
Ser	23.04 ^a^	21.05 ^ab^	15.77 ^b^	1.23	0.039
Tau	17.23	18.71	15.53	0.90	0.371
Thr	30.63	32.04	22.94	1.81	0.083
Tyr	24.01	27.67	24.12	1.27	0.424
Val	31.30 ^a^	31.46 ^a^	21.86 ^b^	1.63	0.017
α-AAA	3.14 ^a^	1.96 ^b^	1.14 ^b^	0.26	0.003
α-ABA	1.84 ^b^	2.90 ^a^	3.15 ^a^	0.19	0.007
β-Ala	1.17	1.00	1.33	0.08	0.281
21 days old					
3-MH	2.12 ^b^	2.85 ^a^	1.46 ^c^	0.15	<0.001
Ala	51.09 ^a^	29.20 ^b^	28.50 ^b^	2.99	<0.001
Arg	23.02 ^a^	16.56 ^b^	12.49 ^b^	1.23	<0.001
Asp	1.52 ^a^	1.24 ^a^	0.56 ^b^	0.11	<0.001
Cit	23.36 ^a^	16.54 ^b^	12.47 ^b^	1.33	0.003
Cys	7.19 ^b^	6.44 ^b^	8.88 ^a^	0.33	0.005
Cysthi	0.89 ^b^	1.69 ^a^	1.90 ^a^	0.14	0.002
ETA	1.00	0.77	0.74	0.09	0.442
Glu	51.94 ^a^	11.99 ^b^	47.63 ^a^	4.09	<0.001
Gly	68.42	57.88	65.07	3.03	0.361
His	13.79 ^a^	12.00 ^a^	7.72 ^b^	0.76	0.002
HYP	26.28 ^a^	12.84 ^b^	13.12 ^b^	1.44	<0.001
Ile	16.18	16.16	16.49	0.78	0.983
Leu	20.49	22.65	23.54	1.31	0.637
Lys	36.47 ^a^	18.89 ^b^	19.79 ^b^	2.35	<0.001
Met	10.19 ^a^	6.39 ^b^	6.18 ^b^	0.59	0.004
Orn	16.54 ^a^	9.00 ^b^	6.41 ^b^	1.10	<0.001
Phe	15.65	14.50	15.15	0.38	0.484
Pro	47.37 ^a^	22.95 ^b^	19.60 ^b^	3.37	<0.001
P-Ser	3.85 ^b^	5.03 ^a^	1.03 ^c^	0.34	<0.001
Sar	1.88	0.83	-	0.57	0.428
Ser	19.71 ^a^	16.02 ^ab^	12.76 ^b^	0.87	0.002
Tau	13.57 ^a^	8.81 ^b^	8.05 ^b^	0.71	0.001
Thr	26.18 ^a^	15.80 ^b^	16.72 ^b^	1.58	0.011
Tyr	22.07 ^a^	13.72 ^b^	15.25 ^b^	1.16	0.004
Val	29.03	31.38	29.61	1.43	0.811
α-AAA	5.93 ^a^	2.09 ^b^	3.37 ^b^	0.45	<0.001
α-ABA	2.08 ^b^	4.91 ^a^	4.41 ^a^	0.38	0.002
β-Ala	1.06	0.91	1.05	0.08	0.714
24 days old					
3-MH	2.52	3.59	3.17	0.23	0.134
Ala	40.45	33.66	38.98	2.70	0.580
Arg	19.34 ^ab^	24.25 ^a^	15.88 ^b^	1.12	0.005
Asp	0.87 ^b^	0.79 ^b^	1.71 ^a^	0.13	0.003
Car	2.77 ^a^	1.22 ^b^	3.08 ^a^	0.25	<0.001
Cit	14.61 ^b^	21.46 ^a^	12.40 ^b^	1.20	0.002
Cys	7.00	7.98	9.36	0.45	0.104
Cysthi	2.34	1.76	2.55	0.17	0.138
ETA	1.19 ^a^	0.48 ^b^	0.65 ^b^	0.10	0.003
Glu	60.66 ^a^	68.47 ^a^	32.8 ^b^	4.86	0.002
Gly	71.98	87.63	81.93	4.92	0.448
His	8.69	9.60	9.03	0.40	0.656
HYP	15.67 ^a^	12.07 ^b^	7.28 ^c^	1.21	0.044
Ile	22.06	23.87	20.97	1.41	0.711
Leu	24.12	29.58	22.86	1.57	0.181
Lys	27.73	29.17	34.77	1.68	0.201
Met	6.27	5.99	7.62	0.30	0.050
Orn	6.36 ^b^	10.06 ^a^	7.16 ^b^	0.53	0.006
Phe	12.92	13.81	14.06	0.53	0.672
Pro	22.46	19.35	21.15	0.81	0.279
P-Ser	2.74 ^b^	4.97 ^a^	1.80 ^b^	0.34	<0.001
Sar	1.11	0.60	-	0.18	0.157
Ser	14.08	17.68	15.97	0.85	0.234
Tau	9.79	7.71	10.71	0.56	0.075
Thr	20.40	24.09	26.31	1.36	0.223
Tyr	11.91	12.87	12.34	0.68	0.854
Val	39.03	31.10	30.60	2.16	0.230
α-AAA	2.14 ^b^	2.10 ^b^	4.61 ^a^	0.35	0.002
α-ABA	4.95	5.46	6.59	0.45	0.344
β-Ala	0.87	1.05	1.23	0.07	0.115

Data are presented as means with their pooled SEM (*n* = 10). ^a,b,c^ Mean values with different letters indicate significant difference (*p* < 0.05). TB piglets, Taoyuan black piglets; XB piglets, Xiangcun black piglets; DR piglets, Duroc piglets. 3-MH, 3-methylhistidine; Ala, alanine; Arg, arginine; Asp, aspartic acid; Car, carnosine; Cit, citrulline; Cys, cystine; Cysthi, cystathionine; ETA, ethanolamine; Glu, glutamic acid; Gly, glycine; His, histidine; HYP, hydroxyl-proline; Ile, isoleucine; Leu, leucine; Lys, lysine; Met, methionine; Orn, L-ornithine; Phe, phenylalanine; Pro, proline; P-Ser, phosphor-serine; Sar, sarcosine; Ser, serine; Tau, taurine; Thr, threonine; Tyr, tyrosine; Val, valine; α-AAA, alpha-aminoadipic acid; α-ABA, alpha-aminobutyric acid; β-Ala, beta-alanine.

**Table 4:** Differences in the jejunal morphology of piglets at different ages.

Item	TB Piglets	XB Piglets	DR Piglets	SEM	*p*-Values
1 days old					
Villus height (μm)	307.68 ^b^	345.73 ^a^	248.91 ^c^	16.51	0.048
Crypt depth (μm)	158.36	162.97	179.21	5.29	0.254
VH/CD	2.03 ^a^	2.23 ^a^	1.40 ^b^	0.12	0.010
10 days old					
Villus height (μm)	306.57	323.86	290.24	13.25	0.591
Crypt depth (μm)	174.86	161.86	177.55	6.52	0.575
VH/CD	1.82	2.14	1.76	0.09	0.160
21 days old					
Villus height (μm)	272.73 ^b^	256.68 ^b^	323.35 ^a^	9.71	0.013
Crypt depth (μm)	168.07 ^b^	158.60 ^b^	206.53 ^a^	6.77	0.008
VH/CD	1.70	1.70	1.60	0.05	0.690
24 days old					
Villus height (μm)	280.35	264.99	294.11	8.60	0.386
Crypt depth (μm)	177.38	167.30	196.26	6.00	0.133
VH/CD	1.62	1.67	1.54	0.04	0.403

Data are presented as means with their pooled SEM (*n* = 10). ^a,b,c^ Mean values with different letters indicate significant difference (*p* < 0.05). TB piglets, Taoyuan black piglets; XB piglets, Xiangcun black piglets; DR piglets, Duroc piglets; VH/CD, villus height/crypt depth ratio.

**Table 5:** Differences in the ileal morphology of piglets at different ages.

Item	TB Piglets	XB Piglets	DR Piglets	SEM	*p*-Values
1 days old					
Villus height (μm)	451.18 ^ab^	602.76 ^a^	325.49 ^b^	39.76	0.010
Crypt depth (μm)	133.19	142.60	124.18	7.68	0.685
VH/CD	3.56 ^a^	4.08 ^a^	2.06 ^b^	0.26	0.004
10 days old					
Villus height (μm)	334.73	353.05	390.26	16.78	0.418
Crypt depth (μm)	100.91 ^b^	118.44 ^ab^	141.54 ^a^	6.46	0.018
VH/CD	3.29	2.88	3.49	0.15	0.223
21 days old					
Villus height (μm)	315.44	279.51	309.20	13.09	0.516
Crypt depth (μm)	124.59	124.26	151.96	6.81	0.165
VH/CD	2.61	2.13	2.13	0.10	0.057
24 days old					
Villus height (μm)	231.71	255.76	270.56	9.62	0.266
Crypt depth (μm)	132.21	129.11	156.88	5.62	0.076
VH/CD	1.83	2.03	1.79	0.07	0.356

Data are presented as means with their pooled SEM (*n* = 10). ^a,b^ Mean values with different letters indicate significant difference (*p* < 0.05). TB piglets, Taoyuan black piglets; XB piglets, Xiangcun black piglets; DR piglets, Duroc piglets; VH/CD, villus height/crypt depth ratio.

**Table 6:** Differences in the jejunal digestive enzyme activities of piglets at different ages (U/g).

Item	TB Piglets	XB Piglets	DR Piglets	SEM	*p*-Values
1 days old					
Amylase	1.62 ^b^	1.19 ^b^	4.40 ^a^	0.48	0.004
Chymase	3.97	4.54	4.28	0.31	0.772
Invertase	13.50 ^ab^	8.99 ^b^	15.76 ^a^	1.13	0.029
Lactase	8.80 ^a^	9.21 ^a^	5.48 ^b^	0.63	0.022
Lipase	0.88	0.58	0.46	0.09	0.138
Maltase	15.62 ^a^	15.98 ^a^	9.04 ^b^	1.08	0.007
Trypsin	3.72 ^a^	2.46 ^b^	0.97 ^c^	0.33	<0.001
10 days old					
Amylase	2.21 ^c^	12.27 ^a^	4.53 ^b^	1.15	<0.001
Chymase	6.69 ^b^	8.21 ^b^	11.89 ^a^	0.69	0.004
Invertase	26.86 ^a^	18.11 ^b^	26.98 ^a^	1.64	0.028
Lactase	11.96	14.09	14.91	0.80	0.333
Lipase	6.65 ^a^	2.71 ^b^	2.17 ^b^	0.49	<0.001
Maltase	27.48	26.80	21.31	1.40	0.207
Trypsin	11.13 ^a^	6.29 ^b^	1.64 ^c^	0.96	<0.001
21 days old					
Amylase	5.22 ^b^	4.36 ^b^	12.72 ^a^	1.05	<0.001
Chymase	12.22	12.03	10.96	0.81	0.806
Invertase	32.78 ^a^	21.98 ^b^	31.58 ^a^	1.73	0.010
Lactase	21.42 ^a^	23.91 ^a^	13.92 ^b^	1.57	0.016
Lipase	2.26 ^ab^	1.43 ^b^	2.68 ^a^	0.21	0.019
Maltase	37.21 ^a^	35.54 ^a^	19.66 ^b^	2.58	0.003
Trypsin	5.74 ^b^	31.30 ^a^	7.86 ^b^	2.83	<0.001
24 days old					
Amylase	5.88 ^b^	5.47 ^b^	11.51 ^a^	0.88	0.002
Chymase	11.65	13.83	11.38	0.66	0.256
Invertase	32.09 ^ab^	40.40 ^a^	26.82 ^b^	2.21	0.037
Lactase	19.63 ^ab^	24.22 ^a^	15.30 ^b^	1.39	0.025
Lipase	2.04	2.85	2.43	0.18	0.193
Maltase	33.85 ^a^	35.13 ^a^	19.86 ^b^	2.10	0.002
Trypsin	6.11 ^b^	23.69 ^a^	9.97 ^b^	2.22	<0.001

Data are presented as means with their pooled SEM (*n* = 8). ^a,b,c^ Mean values with different letters indicate significant difference (*p* < 0.05). TB piglets, Taoyuan black piglets; XB piglets, Xiangcun black piglets; DR piglets, Duroc piglets.

**Table 7:** Differences in the ileal digestive enzyme activities of piglets at different ages (U/g).

Item	TB Piglets	XB Piglets	DR Piglets	SEM	*p*-Values
1 days old					
Amylase	3.96 ^b^	2.47 ^b^	11.05 ^a^	1.01	<0.001
Chymase	5.34 ^b^	7.56 ^b^	19.67 ^a^	1.65	<0.001
Invertase	12.91 ^b^	8.19 ^b^	28.25 ^a^	2.46	<0.001
Lactase	6.59 ^c^	15.17 ^b^	30.91 ^a^	2.36	<0.001
Lipase	1.29 ^b^	1.97 ^b^	5.10 ^a^	0.41	<0.001
Maltase	8.69 ^c^	20.24 ^b^	50.45 ^a^	4.11	<0.001
Trypsin	5.28 ^b^	4.50 ^b^	16.52 ^a^	1.41	<0.001
10 days old					
Amylase	11.69	7.87	7.00	0.90	0.053
Chymase	12.98 ^b^	22.15 ^a^	21.05 ^a^	1.47	0.009
Invertase	42.52	36.38	29.26	2.40	0.082
Lactase	14.53 ^b^	48.20 ^a^	54.23 ^a^	5.92	0.007
Lipase	3.53 ^b^	6.33 ^a^	6.55 ^a^	0.53	0.018
Maltase	19.59 ^b^	67.68 ^a^	76.44 ^a^	7.19	<0.001
Trypsin	14.77	16.41	10.98	1.12	0.145
21 days old					
Amylase	17.64 ^a^	1.88 ^c^	12.03 ^b^	1.67	<0.001
Chymase	21.26 ^a^	13.08 ^b^	24.69 ^a^	1.55	0.003
Invertase	58.42 ^a^	23.26 ^b^	51.82 ^a^	5.41	0.012
Lactase	22.00 ^b^	22.76 ^b^	65.61 ^a^	5.17	<0.001
Lipase	4.56 ^ab^	3.04 ^b^	6.32 ^a^	0.46	0.008
Maltase	32.78 ^b^	37.45 ^b^	78.85 ^a^	5.07	<0.001
Trypsin	18.22 ^a^	4.61 ^b^	20.26 ^a^	1.91	<0.001
24 days old					
Amylase	17.61 ^a^	6.52 ^b^	21.19 ^a^	1.45	<0.001
Chymase	16.37 ^b^	14.92 ^b^	26.89 ^a^	1.47	<0.001
Invertase	46.25 ^a^	34.69 ^b^	45.66 ^a^	1.97	0.018
Lactase	20.46 ^b^	27.43 ^b^	51.79 ^a^	3.25	<0.001
Lipase	5.01 ^b^	2.47 ^c^	7.99 ^a^	0.56	<0.001
Maltase	27.28 ^c^	44.55 ^b^	77.03 ^a^	5.03	<0.001
Trypsin	21.15 ^a^	10.85 ^b^	23.92 ^a^	1.66	<0.001

Data are presented as means with their pooled SEM (*n* = 8). ^a,b,c^ Mean values with different letters indicate significant difference (*p* < 0.05). TB piglets, Taoyuan black piglets; XB piglets, Xiangcun black piglets; DR piglets, Duroc piglets.

## Data Availability

Not applicable.

## Supplementary Materials

The following supporting information can be downloaded at: https://www.mdpi.com/article/10.3390/metabo13010132/s1, Table S1: Primer sequences of the target genes.

## References

[1] Hao Y., Wang J., Teng D., Wang X., Mao R.Y., Na Y., Ma X. (2020). A prospective on multiple biological activities of lactoferrin contributing to piglet welfare. Biochem. Cell Biol..

[2] Mace O.J., Marshall F. (2013). Digestive physiology of the pig symposium: Gut chemosensing and the regulation of nutrient absorption and energy supply. J. Anim. Sci..

[3] Weström B., Arévalo Sureda E., Pierzynowska K., Pierzynowski S.G., Pérez–Cano F.J. (2020). The immature gut barrier and its importance in establishing immunity in newborn mammals. Front. Immunol..

[4] Wang L.X., Zhu F., Li J.Z., Li Y.L., Ding X.Q., Yin J., Xiong X., Yang H.S. (2020). Epidermal growth factor promotes intestinal secretory cell differentiation in weaning piglets via Wnt/β-catenin signalling. Animal.

[5] Chen Y., Zhang H., Chen Y., Jia P., Ji S., Zhang Y., Wang T. (2021). Resveratrol and its derivative pterostilbene ameliorate intestine injury in intrauterine growth–retarded weanling piglets by modulating redox status and gut microbiota. J. Anim. Sci. Biotechnol..

[6] Canario L., Lundgren H., Haandlykken M., Rydhmer L. (2010). Genetics of growth in piglets and the association with homogeneity of body weight within litters. J. Anim. Sci..

[7] Tang Z., Fu Y., Xu J., Zhu M., Li X., Yu M., Zhao S., Liu X. (2020). Discovery of selection–driven genetic differences of Duroc, Landrace, and Yorkshire pig breeds by EigenGWAS and *F_st_* analyses. Anim. Genet..

[8] Barea R., Nieto R., Vitari F., Domeneghini C., Aguilera J.F. (2011). Effects of pig genotype (Iberian *v.* Landrace × Large White) on nutrient digestibility, relative organ weight and small intestine structure at two stages of growth. Animal.

[9] Bergamaschi M., Tiezzi F., Howard J., Huang Y.J., Gray K.A., Schillebeeckx C., McNulty N.P., Maltecca C. (2020). Gut microbiome composition differences among breeds impact feed efficiency in swine. Microbiome.

[10] Picone G., Zappaterra M., Luise D., Trimigno A., Capozzi F., Motta V., Davoli R., Costa L.N., Bosi P., Trevisi P. (2018). Metabolomics characterization of colostrum in three sow breeds and its influences on piglets’ survival and litter growth rates. J. Anim. Sci. Biotechnol..

[11] Christensen B., Huber L.A. (2022). The effects of creep feed composition and form and nursery diet complexity on small intestinal morphology and jejunal mucosa-specific enzyme activities after weaning in pigs. J. Anim. Sci..

[12] Saqui–Salces M., Huang Z., Vila M.F., Li J., Mielke J.A., Urriola P.E., Shurson G.C. (2017). Modulation of intestinal cell differentiation in growing pigs is dependent on the fiber source in the diet. J. Anim. Sci..

[13] Azad M.A.K., Gao Q., Ma C., Wang K., Kong X. (2022). Betaine hydrochloride addition in Bama mini–pig’s diets during gestation and lactation enhances immunity and alters intestine microbiota of suckling piglets. J. Sci. Food Agric..

[14] Feng Z., Guo J.P., Kong X., Wang W., Li F.N., Nyachoti M., Yin Y.L. (2012). Molecular cloning and expression profiling of G protein coupled receptor 120 in Landrace pig and different Chinese indigenous pig breeds. J. Food Agric. Environ..

[15] Brossard L., Nieto R., Charneca R., Araujo J.P., Pugliese C., Radović Č., Čandek–Potokar M. (2019). Modelling nutritional requirements of growing pigs from local breeds using InraPorc. Animals.

[16] Diao S.Q., Xu Z.T., Ye S.P., Huang S.W., Teng J.Y., Yuan X.L., Chen Z.M., Zhang H., Li J.Q., Zhang Z. (2021). Exploring the genetic features and signatures of selection in South China indigenous pigs. J. Integr. Agric..

[17] Wang M., Yang C., Wang Q.Y., Li J.Z., Li Y.L., Ding X.Q., Yin J., Yang H.S., Yin Y.L. (2020). The growth performance, intestinal digestive and absorptive capabilities in piglets with different lengths of small intestines. Animal.

[18] Elefson S.K., Lu N., Chevalier T., Dierking S., Wang D., Monegue H.J., Matthews J.C., Jang Y.D., Chen J., Rentfrow G.K. (2021). Assessment of visceral organ growth in pigs from birth through 150 kg. J. Anim. Sci..

[19] Czech A., Nowakowicz-Debek B., Łukaszewicz M., Florek M., Ossowski M., Wlazło Ł. (2022). Effect of fermented rapeseed meal in the mixture for growing pigs on the gastrointestinal tract, antioxidant status, and immune response. Sci. Rep..

[20] Liu Y., Kong X., Jiang G., Tan B., Deng J., Yang X., Li F., Xiong X., Yin Y. (2015). Effects of dietary protein/energy ratio on growth performance, carcass trait, meat quality, and plasma metabolites in pigs of different genotypes. J. Anim. Sci. Biotechnol..

[21] Cheng Y., Song M., Zhu Q., Azad M.A.K., Gao Q., Kong X. (2021). Dietary betaine addition alters carcass traits, meat quality, and nitrogen metabolism of Bama mini-pigs. Front. Nutr..

[22] Zhu Q., Xie P., Li H., Blachier F., Yin Y., Kong X. (2021). Dynamic changes of metabolite profiles in maternal biofluids during gestation period in Huanjiang mini-pigs. Front. Vet. Sci..

[23] Cheng Y., Song M., Zhu Q., Azad M.A.K., Gao Q., Kong X. (2021). Impacts of betaine addition in sow and piglet’s diets on growth performance, plasma hormone, and lipid metabolism of Bama mini–pigs. Front. Nutr..

[24] Song C., Jiang J., Han X., Yu G., Pang Y. (2014). Effect of immunological stress to neuroendocrine and gene expression in different swine breeds. Mol. Biol. Rep..

[25] Nørgaard J., Florescu I., Krogh U., Nielsen T. (2021). Amino acid absorption profiles in growing pigs fed different protein sources. Animals.

[26] McGilvray W.D., Klein D., Wooten H., Dawson J.A., Hewitt D., Rakhshandeh A.R., de Lange C.F.M., Rakhshandeh A. (2019). Immune system stimulation induced by *Escherichia coli* lipopolysaccharide alters plasma free amino acid flux and dietary nitrogen utilization in growing pigs. J. Anim. Sci..

[27] Ding X., Li H., Wen Z., Hou Y., Wang G., Fan J., Qian L. (2020). Effects of fermented tea residue on fattening performance, meat quality, digestive performance, serum antioxidant capacity, and intestinal morphology in fatteners. Animals.

[28] Walsh A., Sweeney T., Bahar B., Flynn B., O’Doherty J. (2012). The effect of chitooligosaccharide supplementation on intestinal morphology, selected microbial populations, volatile fatty acid concentrations and immune gene expression in the weaned pig. Animal.

[29] Fan C.L., Han X.Y., Xu Z.R., Wang L.J., Shi L.R. (2009). Effects of β-glucanase and xylanase supplementation on gastrointestinal digestive enzyme activities of weaned piglets fed a barley-based diet. J. Anim. Physiol. Anim. Nutr..

[30] Tian Z.M., Ma X.Y., Yang X.F., Fan Q.L., Xiong Y.X., Qiu Y.Q., Wang L., Wen X.L., Jiang Z.Y. (2016). Influence of low protein diets on gene expression of digestive enzymes and hormone secretion in the gastrointestinal tract of young weaned piglets. J. Zhejiang Univ. B.

[31] Chin A.M., Hill D.R., Aurora M., Spence J.R. (2017). Morphogenesis and maturation of the embryonic and postnatal intestine. Semin. Cell Dev. Biol..

[32] Maljaars P., Peters H., Mela D., Masclee A. (2008). Ileal brake: A sensible food target for appetite control. A review. Physiol. Behav..

[33] Zhang J., Zhao D., Yi D., Wu M., Chen H., Wu T., Zhou J., Li P., Hou Y., Wu G. (2019). Microarray analysis reveals the inhibition of intestinal expression of nutrient transporters in piglets infected with porcine epidemic diarrhea virus. Sci. Rep..

[34] Röder P.V., Geillinger K.E., Zietek T.S., Thorens B., Koepsell H., Daniel H. (2014). The role of SGLT1 and GLUT2 in intestinal glucose transport and sensing. PLoS ONE.

[35] Yang H.S., Fu D.Z., Kong X.F., Wang W.C., Yang X.J., Nyachoti C.M., Yin Y.L. (2013). Dietary supplementation with N-carbamylglutamate increases the expression of intestinal amino acid transporters in weaned Huanjiang mini-pig piglets. J. Anim. Sci..

[36] Ma J., Duan Y., Li R., Liang X., Li T., Huang X., Yin Y., Yin J. (2022). Gut microbial profiles and the role in lipid metabolism in Shaziling pigs. Anim. Nutr..

[37] Vigors S., Sweeney T., O’Shea C.J., Kelly A.K., O’Doherty J.V. (2016). Pigs that are divergent in feed efficiency, differ in intestinal enzyme and nutrient transporter gene expression, nutrient digestibility and microbial activity. Animal.

[38] Wang M., Yang C., Wang Q., Li J., Huang P., Li Y., Ding X., Yang H., Yin Y. (2019). The relationship between villous height and growth performance, small intestinal mucosal enzymes activities and nutrient transporters expression in weaned piglets. J. Anim. Physiol. Anim. Nutr..

