# Supplementary material for: Comparison of the Pig Breeds in the Small Intestinal Morphology and Digestive Functions at Different Ages
Source: Metabolites. 2023 Jan 15;13(1):132. doi: 10.3390/metabo13010132 (PMC9863662; doi:10.3390/metabo13010132)
Supplement: Supplementary file 1 [file metabolites-13-00132-s001.zip › metabolites-2105228-supplementary.pdf]

Supplementary Table S1. Primer sequences of the target genes

Target Genes	Accession No.	Primer Sequences (5'-3')
FABP1	NM_001004046.2	F: TGAACTCAACGGTGACATA
		R: ATTCTCTTGCTGATTCTCTTG
FABP2	NM_001031780.1	F: CAGCCTCGCAGACGGAACTGAA
		R: GTGTTCTGGGCTGTGCTCCAAGA
FABP4	NM_001002817.1	F: TGGAAACTTGTCTCCAGTG
		R: GGTACTTTCTGATCTAATGGTG
GAPDH	NM_001206359.1	F: CAGCAATGCCTCCTGTACCA
		R: ACGATGCCGAAGTTGTCATG
GLUT1	XM_003482115.1	F: TGCTCATCAACCGCAATGA
		R: GTTCCGCGCAGCTTCTTC
GLUT2	NM_001097417.1	F: CCAGGCCCCATCCCCTGGTT
		R: GCGGGTCCAGTTGCTGAATGC
GLUT5	XM_021095282.1	F: CCCAGGAGCCGGTCAAG
		R: TCAGCGTCGCCAAAGCA
PEPT1	NM_214347.1	F: GGATAGCCTGTACCCCAAGCT
		R: CATCCTCCACGTGCTTCTTGA
SGLT1	NM_001164021.1	F: GGCTGGACGAAGTATGGTG
		R: ACAACCACCCAAATCAGAGC
SLC1A1	NM_001164649.1	F: GGCACCGCACTCTACGAAGCA
		R: GCCCACGGCACTTAGCACGA
SLC7A7	NM_001110421.1	F: TCAAGTGGGGAACCCTGGTA
		R: ATGGAGAGGGGCAGATTCCT
SLC6A19	XM_003359855.4	F: TCTGTCCACAACAACTGCGAG
		R: CAGCGAAGTTCTCCTGCGTC

GLUT, glucose transporter; *SGLT1*, sodium-glucose linked transporter 1; *PEPT1*, peptide transporter 1; *SLC1A1*, solute carrier family 1 member 1; *SLC7A7*, solute carrier family 7 member 7; *SLC6A19*, sodium-dependent neutral amino acid transporter; *SLC1A5*, solute carrier family 1 member 5; *SLC7A9*, solute carrier family 7 member 9; *FABP*, fatty acid binding protein.
